# Mechanical writing of n-type conductive layers on the SrTiO_3_ surface in nanoscale

**DOI:** 10.1038/srep10841

**Published:** 2015-06-04

**Authors:** Yuhang Wang, Kehan Zhao, Xiaolan Shi, Geng Li, Guanlin Xie, Xubo Lai, Jun Ni, Liuwan Zhang

**Affiliations:** 1 State Key Laboratory of Low-Dimensional Quantum Physics, Collaborative Innovation Center of Quantum Matter, Department of Physics, Tsinghua University, 100084, Beijing, China; 2 National Key Laboratory of Shock Wave and Detonation Physics, Institute of Fluid Physics, China Academy of Engineering Physics, 621999, Mianyang, Sichuan, China; 3 Department of Physics, Beijing University of Aeronautics and Astronautics, 100191, Beijing, China

## Abstract

The fabrication and control of the conductive surface and interface on insulating SrTiO_3_ bulk provide a pathway for oxide electronics. The controllable manipulation of local doping concentration in semiconductors is an important step for nano-electronics. Here we show that conductive patterns can be written on bare SrTiO_3_ surface by controllable doping in nanoscale using the mechanical interactions of atomic force microscopy tip without applying external electric field. The conductivity of the layer is n-type, oxygen sensitive, and can be effectively tuned by the gate voltage. Hence, our findings have potential applications in oxide nano-circuits and oxygen sensors.

In recent years, the conductive properties of SrTiO_3_ (STO) surface and interface have been widely studied since the first discovery of the two dimensional electron gas (2DEG) at the LaAlO_3_ (LAO)/STO interface^1^, which reveals various interesting properties including metal-insulator transition (MIT)^2^,^3^, ferromagnetism^4^,^5^,^6^, superconductivity^7^,^8^ and even the coexistence of them^9^. However, the mechanism of this conductive surface layer is still debating. The contribution from the oxygen vacancy cannot be definitely precluded although the “polar catastrophe” scenario is widely accepted^10^,^11^. In order to elucidate the real mechanism, the conductive behavior of STO surface was investigated in a number of similar systems including LaVO_3_/STO interface^12^, ionic liquid gated STO^13^,^14^ and even bare STO surface^15^,^16^,^17^,^18^,^19^. The electric field induced conductivity at the ionic liquid-gated STO surface was considered as a strong support for the “polar catastrophe” scenario^13^. However, Mingyang Li *et al*. have shown that the gating effect was suppressed in oxygen atmosphere, which was in favor of the oxygen vacancy scenario^14^. Furthermore, conductive layers could be formed at bare STO surface by many different kinds of methods. The metallic surface states at the H-adsorbed STO surface was predicted by DFT calculations^15^ and was then realized by later experiments^16^. Ion bombarded^17^ and UV irradiated^18^ STO surface in vacuum were also demonstrated to be metallic. Santander-Syro reported the existence of 2DEG on simply vacuum fractured STO surface, which shows similar electronic properties to the LAO/STO interface^19^. Here the 2DEG is attributed to the 1D electric confinement produced by the surface positive oxygen vacancies. The quantum confinement causes a splitting of the degenerate d_xy_, d_yz_, d_xz_ sub-bands of the Ti ions, giving rise to a downshift of the conduction band across the Fermi level. These results show that the surface oxygen vacancy can produce a conductive or even metallic layer at STO surface without external electric field.

One of the key features of semiconductors for technological applications is the possibility of controllable doping in nanoscale^20^. Using a biased conductive atomic force microscopy (AFM) tip, the LAO/STO interface can be reversibly switched between metallic and insulating state microscopically, showing the effect of external electric field^21^,^22^. In this article, we report the writing of n-type conductive nanolayers on the bare STO surface using contact AFM tip without applying external electric field. Unlike the metallic behavior of the vacuum fractured STO surface, the temperature dependence of the conductivity of this nanolayer shows a semiconducting thermal activation behavior. Moreover, the conductivity of the nanolayer can be tuned by a back gate voltage, and erased by the oxygen absorption. These features make it possible to fabricate oxygen sensitive quantum dots, conductive patterns or even electronic circuits in nanoscale on oxide semiconductor surfaces.

## Results

### Conductive layer fabrication

**Figure 1:**
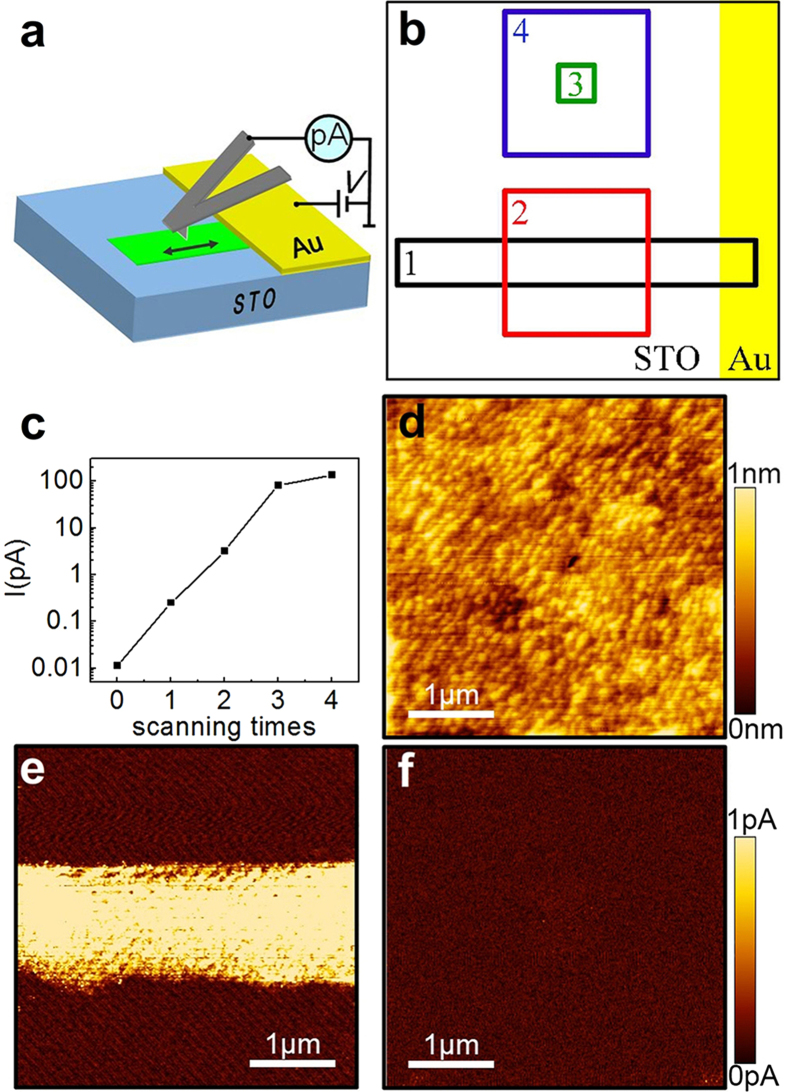
Conductive surface layer fabrication and verification. (**a**) AFM writing and conductivity measurement configuration. (**b**) Schematic illustration for the writing and measurement areas. (**c**) Tip current under 5 V bias (at the center of the black rectangle 1 in Fig. 1b) versus scanning times. (**d**) Topography of the STO surface after scratching (red square 2 in Fig. 1b). (**e**) Current distribution image over the area of the red square 2 in Fig. 1b. The bright stripe corresponds to the scratched conductive layer connecting the electrode. (**f**) Current distribution image over the area of blue square 4 in Fig. 1b. No current is detected over the scratched area (green square 3 in Fig. 1b) isolated from the electrode.

The sample was prepared by depositing 200 nm thick gold film stripe on the TiO_2_-terminated atomically flat (001) STO single crystal substrate. The measurements are carried out in a scanning probe microscopy (SPM) vacuum chamber with a base pressure of 1 × 10^−4^ Pa. A doped diamond coated conductive tip was used to fabricate the conductive nanolayer, image the topography, and measure the local conductivity. The applied pressure under the tip is ~1 GPa and ~10 MPa for conductive layer fabrication and conductivity imaging, respectively. As schematically shown in Fig. 1a, the tip is grounded (through a picoammeter) and a voltage V is applied to the Au electrode.

The scanning and measuring diagram is illustrated in Fig. 1a,b. First, we scanned a 10 μm × 1 μm area across the Au/STO border (black rectangle 1 in Fig. 1b) line by line using contact mode four times with a tip pressure of ~1 GPa. The scanning direction is along the long side of the rectangle. There are 64 lines over the rectangular area. The distance between neighboring lines is ~15.6 nm, much shorter than the radius of the tip (~35 nm). During this scratching process, the voltage between the tip and electrode was kept 0 V. After each scanning, the tip was fixed at the center of the area and the current between the tip and Au electrode was measured under a bias of 5 V. As plotted in Fig. 1c, the measured current was almost exponentially increased with scanning times until the internal picoammeter is saturated (the upper current limit is 100 pA). This exponential dependence is further discussed in the **Discussion** section. After this scanning process, the topography (Fig. 1d) and current distribution (Fig. 1e) over a 4 μm × 4 μm area (red square 2 in Fig. 1b) were imaged under a bias of 2 V using the Sampling Intelligent Scan (SIS) mode. As seen from Fig. 1d the step-terrace structure on the STO surface keeps continuous at the border between the scratched and unscratched area. No visible change in the surface structure was observed after the scratching process. However, as shown in Fig. 1e, the strong conductivity only occurs on the scratched area, while the current from the unscratched area is nearly beyond the instrument lower limit. This indicates that the high conductivity is resulted from the mechanical scratching process and has nothing to do with the electric measurement process.

Previously, Szot *et al*. reported that the dislocation networks are threaded across the whole STO single crystal^23^. Yoshida *et al*. proposed that the dielectric properties of the surface layer of STO single crystal were much different from the bulk^24^. One possibility is that there might exist conductive dislocation filament networks below a thin insulating surface layer in STO. Once the insulating surface layer is removed off by the tip scratching, the conductive filament is exposed. As a result the conductive tip is electrically connected to the electrode through the filament. In order to rule out this possibility, we scanned a 1 μm × 1 μm area (green square 3 in Fig. 1b) in contact mode 4 times, which is not connected to the Au electrode. Then the conductivity distribution is recorded over a 4 μm × 4 μm area (blue square 4 in Fig. 1b) under 2 V bias in SIS mode. No detectable current was observed in either scratched or unscratched area, which implies that the conductive area observed in Fig. 1e is confined at the tip-stress induced surface region.

### Transport properties characterization

**Figure 2:**
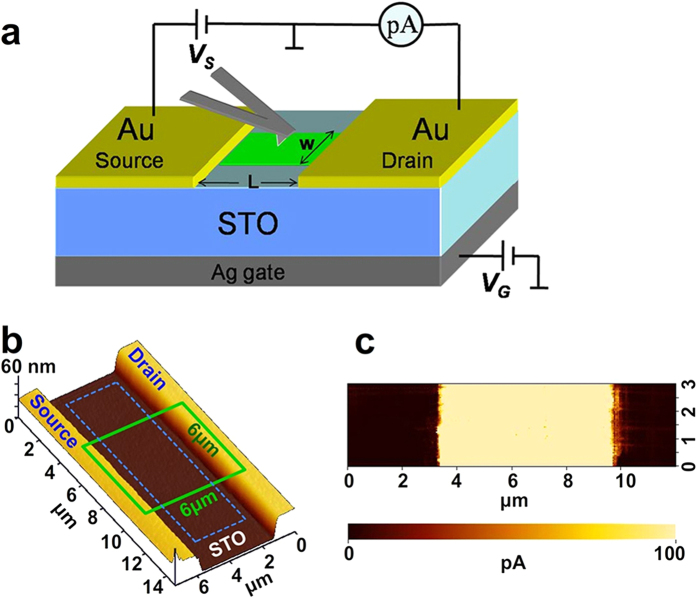
Conductive surface layer on the FET sample. (**a**) Schematic illustration for the FET measurement configuration. (**b**) Topography of the FET sample surface. (**c**) Current distribution of the conductive nanolayer (blue rectangle in b).

The transport properties of the conductive layer were further investigated using a field effect transistor (FET) configuration as depicted in Fig. 2a. The corresponding surface topography is shown in Fig. 2b. Two Au stripes, 5 μm apart, were fabricated on the STO surface by lithography technique as source and drain electrodes. Silver paint was spread at the back side of the wafer as the gate electrode. A 6 μm × 6 μm square area (green square in Fig. 2b) connecting the source and drain electrodes was scanned repeatedly in contact mode following the same scratching process mentioned above. Then the conductivity distribution under 2 V bias over a 12 μm × 3 μm area (blue dotted rectangle in Fig. 2b) is measured in SIS mode as shown in Fig. 2c. It can be confirmed that a 6 μm wide conductive channel between the source and drain was formed.

**Figure 3:**
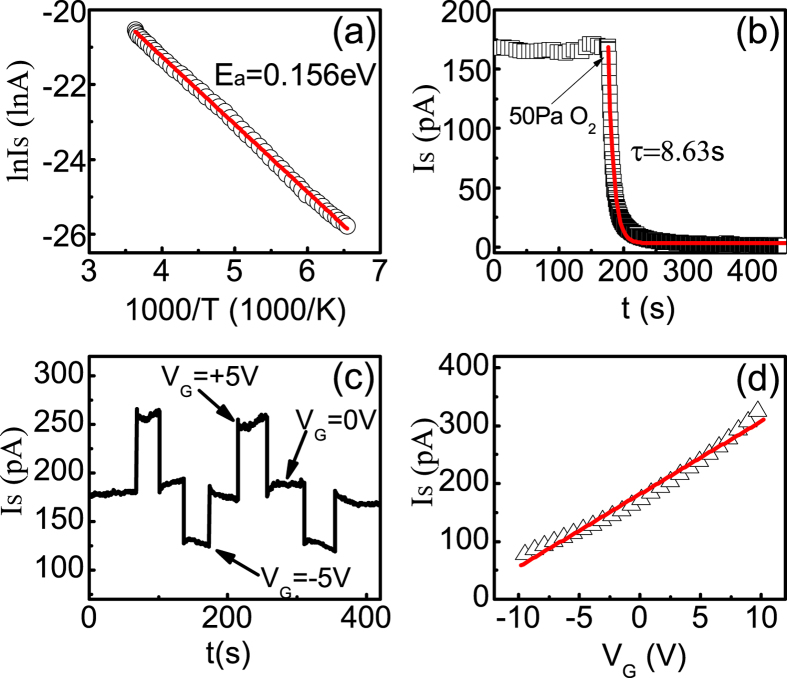
Transport properties of the conductive layer. (**a**) The dependence of the conductivity on temperature. (**b**) The response of the conductivity to the exposure of oxygen gas. (**c**) The surface conductivity tuned by back gate voltage. (**d**) The source-drain current as a function of the gate voltage. The red curves in (**a**), (**b**) and (**d**) are fitting results.

The temperature dependence of the conductivity of the nanolayer was then measured. A voltage of V_S_ = 3 V was applied between the source and drain, while the gate voltage V_G_ was kept 0 V. The source current I_S_ as a function of temperature is plotted in Fig. 3a. The current decreases with decreasing temperature, following the Arrhenius relationship


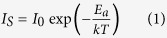


where Ea is the activation energy, k the Boltzmann’s constant, T the absolute temperature and I_0_ the pre-exponential factor. The fitted E_a_ is 0.156 eV, close to the first ionization energy of oxygen vacancy in perovskite oxides^25^. This suggests that the induced conductivity is closely related to the surface oxygen vacancy. To verify this suggestion, we fill the SPM vacuum chamber with oxygen gas to 50 Pa at room temperature. With the conductive surface exposed to oxygen, the source current I_S_ drops exponentially to zero, following the form of 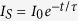 as shown in Fig. 3b. The decay time constant τ is fitted to be 8.63 s. The source current I_S_ remains zero after the previous high vacuum is returned. Re-scratching the surface in vacuum could restore the conductivity. These results strongly implied that the observed conductivity originated from surface doping of oxygen vacancy generated by the tip scratching in vacuum. The re-oxidation of the scratched surface in oxygen destroys the conductivity. Since ion-beam etching and vacuum-cleaving could produce oxygen vacancy on STO surface^17^,^18^,^19^, it is reasonable to believe that AFM tip scanning with high pressure in vacuum could knock out the oxygen atoms from the surface as well.

Figure 3c demonstrates the manipulation of the source current I_S_ by the gate voltage V_G_. Under a constant source-drain voltage of 2 V, positive gate voltage increases Is, while negative gate voltage decreases Is. For the FET configuration, +5 V (-5 V) back gate voltage will cause the accumulation (depletion) of electrons at the surface^20^. The polarity dependence indicates that the conductive surface layer is n-type and the majority carrier is electron, in accordance with the oxygen vacancy scenario. The dependence of I_S_ on V_G_ under a constant source voltage V_S_ = 2 V is plotted in Fig. 3d. The I_S_- V_G_ curve shows an approximate linear behavior, in accordance with the standard transfer characteristics of FETs. According to the FET model, the source current Is is expressed as


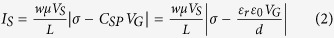


where μ is the carrier mobility in the surface layer, σ the carrier areal density, C_SP_ specific capacitance between the conductive surface layer and the gate, ε_r_ the relative permittivity of STO, ε_0_ vacuum permittivity, d the thickness of STO, w and L the width and length of the conductive surface layer^26^. Taking w = 6 μm, L = 5 μm, ε_r_ = 300, the mobility μ and carrier areal density σ are estimated to be 0.01 cm^2^V^−1^s^−1^ and 4.786 × 10^10^ cm^−2^, respectively, from the fitted slop and intercept of the I_S_-V_S_ curve.

## Discussion

**Figure 4:**
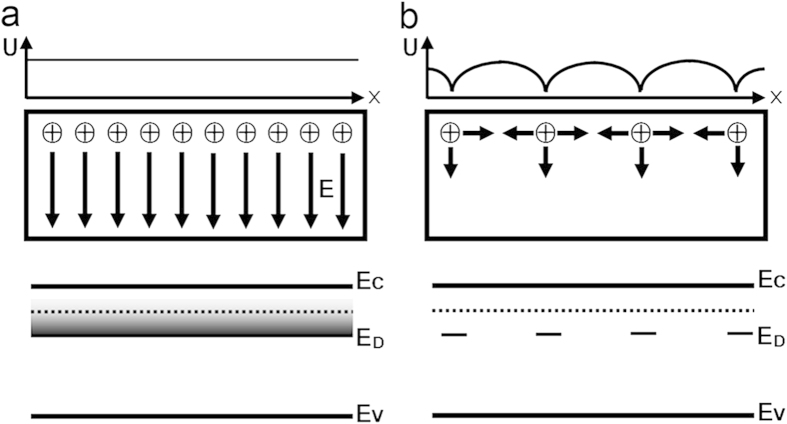
The oxygen vacancy density effects. The in-plane potential profile (upper), oxygen vacancy distribution (middle) and band diagram (bottom) with (**a**) Dense surface oxygen vacancy and (**b**) Sparse surface oxygen vacancy. E_C_, E_V_ and E_D_ denote conduction band, valence band and oxygen vacancy donor level, respectively. The dotted lines denote the Fermi level.

It is worth noticing that our scratched surface layer is semiconducting, quite different from the metallic behavior of vacuum fractured STO surface^19^, although the conductivity is all supposed to result from the n-type doping of oxygen vacancy. The critical difference is that the carrier density of our scratched surface layer is roughly four orders of magnitude smaller than that of the vacuum cleaved surface. That is, the amount of the oxygen vacancies of the vacuum cleaved surface is almost four orders of magnitude larger than that of our surface. In order to make it more clear, the distribution of the oxygen vacancies, potential profile, and the band diagram are schematically illustrated in Fig. 4. For convenience, the surface plane is defined as x-y plane with z direction perpendicular to the surface. For vacuum cleaved surface as shown in Fig. 4a, the areal density of the oxygen vacancies is as high as 1.1 × 10^14^ cm^−2^, forming a homogenous positively charged layer on the surface. This charged layer will generate a uniform electric field perpendicular to the surface, leading to an electric potential well along z direction. However, in the surface plane, the electric potential profile is nearly flat due to the high density of the oxygen vacancy. According to Santander-Syro *et al*.^19^ the one dimensional (1D) quantum confinement causes the splitting and downshift of the conduction band across the Fermi level and results in a metallic 2DEG. The oxygen vacancies are accumulated on the top surface of the vacuum fractured STO. While the resulting delocalized electrons would penetrate into a depth which is comparable to the width of the potential well across the Fermi level. Due to the well separation in space between the delocalized carriers and the donors, the electrons have a higher mobility (7 cm^2^V^−1^s^−1^ at 295 K, >3000 cm^2^V^−1^s^−1^ at 3 K).

In contrast, the areal density of oxygen vacancy on the AFM tip scratched surface is only 4.8 × 10^10^ cm^−2^. As schematically shown in Fig. 4b, a hydrogen-like central field exists in the vicinity of oxygen vacancy due to the large distance between neighboring oxygen vacancies (approximately 46 nm on average). In this case the conduction electrons are mainly localized in the potential wells centered at the oxygen vacancies. The conduction electrons hopping from one oxygen vacancy to its neighboring site along the surface plane have to overcome a potential barrier U_0_. Considering two neighboring oxygen vacancies separated by a distance of d, the barrier height U_0_ can be roughly expressed from Coulomb’s law as 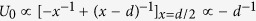. If we assume that one trace of scratching along one line produces the same amount of oxygen vacancies, the distance between neighboring oxygen vacancies after n times scratching becomes 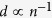. As a result, 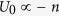. According to the thermal activation model, the source current follows the Arrhenius relationship 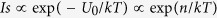. The exponential dependence of the surface current on the scratching times was indeed observed in Fig. 1c. This kind of hopping motion of electrons is also consistent with the observed semiconducting conduction behavior (Fig. 3a) with a low mobility.

In summary, n-type conductive surface layers were mechanically written on STO surface by AFM tip without applying external electric field. The conductivity of the surface layer is sensitive to oxygen, and can be effectively tuned by the gate voltage. Our findings show the possibility of controllable doping at STO surface in nanoscale and have potential applications in nano-circuit and oxygen sensors at oxide surface.

## Methods

### Sample preparation

The commercially available (001)-oriented one-side polished STO single crystal (MTI, miscut < 0.5°) was used. The wafer was rinsed subsequently with acetone, ethanol, and deionized water in an ultrasonic bath (40 kHz, 70 W) for 3 minutes. Then the STO wafer was annealed in flowing high purity O_2_ (99.999%) at 800 °C for 5 h to obtain a clean flat stepped surface and eliminate any possible oxygen vacancy in the bulk. The treated STO surface is predominantly terminated with TiO_2_ atomic plane. A gold film stripe of 200 nm thick was then deposited on the treated surface through a mask by DC magnetron sputtering at room temperature under Ar pressure of 1 Pa. This sample was used for the conductive layer fabrication section (Fig. 1a). To further investigate the transport properties of the conductive layer, an FET sample was fabricated as shown in Fig. 2a. Two gold film electrodes, 5 μm apart, were fabricated using the standard lift-off photolithography technique on the STO surface. Silver paint was spread at the back side of the wafer as the gate electrode (Fig. 2).

### Conductive atomic force microscopy

The experiments were carried out in a SPM chamber (SII E-sweep) under a base pressure of 1 × 10^−4^ Pa. A doped diamond coated conductive tip (DDESP-10, Bruker Company) was used to fabricate the conductive layer, image the topography, and measure the local conductivity. The conductive surface layer was fabricated by scratching the STO surface line by line using the contact mode. The applied pressure under the tip is ~1 GPa and the scanning speed is 5 μm/s for conductive layer fabrication. During this scratching process, no voltage was applied between the tip and electrode.

After the whole designated region was scratched, the conductivity distribution on an area including part of the scratched surface was continuously imaged (measured) under a bias of 2 V. To avoid additional scratching on the surface during the current imaging process, a lower tip pressure (10 MPa) was applied and the Sampling Intelligent Scan (SIS) mode was used. In the SIS mode, the tip is contacted to the surface only when measuring a pixel. After one pixel is measured, the tip is lifted upward off the surface for about 50 nm, moved above the next pixel, lowered to contact the surface and makes another measurement. In this way, the mechanical influence on the surface during the current measuring process can be negligible.

### Electric measurement

The current image between the tip and the Au electrode was measured by an internal picoammeter of the SPM under the internal voltage bias. The positive bias was defined by the current flowing from the Au electrode through the STO surface to the tip (Fig. 1a). The saturation current of the picoammeter is 100 pA and the sensitivity is 100 fA. During the transport measurement of the FET sample, the tip was disconnected from the internal circuit. The source-drain current Is was measured by a Keithley 6517 electrometer, and the gate voltage V_G_ was applied by Keithley 2400 source meter as shown in Fig. 2a.

## Additional Information

**How to cite this article**: Wang, Y. *et al*. Mechanical writing of n-type conductive layers on the SrTiO_3_ surface in nanoscale. *Sci. Rep*. **5**, 10841; doi: 10.1038/srep10841 (2015).
